# Ultrasound examination assisted clinical diagnosis of Leydig cell tumor of ovary: An extremely rare case report

**DOI:** 10.1097/MD.0000000000032451

**Published:** 2022-12-30

**Authors:** Cai Tian, Xiaodong Li, Zijie Fu, Yiwei Han, Jingqiao Liu

**Affiliations:** a The First Hospital of Hebei Medical University.

**Keywords:** case report, Leydig cell tumor, ovary tumor, transvaginal ultrasound

## Abstract

Leydig cell tumor (LCT) is a sex cord-stromal tumor, which is a clinically rare ovarian tumor. It is characterized by endocrine hormonal changes and usually occurs in postmenopausal women.

**Patient concerns:**

We report the clinical case of a 38-year-old female of childbearing age with LCT of the right ovary who presented with significantly decreased menstrual flow and elevated androgen levels, with persistent hypoechoic areas in the ovary as demonstrated by transvaginal ultrasound.

**Diagnosis:**

The transvaginal ultrasound suggested the presence of a hypoechoic area in the right ovary with elevated androgens, interstitial tumor of the ovarian sex cord may be considered.

**Interventions:**

The patient underwent laparoscopic right adnexectomy.

**Outcomes:**

Postoperative pathology confirmed the morphology and immunohistochemistry of the right adnexa consistent with LCT, and no areas of malignant transformation were found on multiple sections of the surgical specimen. The patient had normal androgen levels at postoperative day 2, day 45 and month 3. There was no sign of recurrence.

**Conclusion:**

This case suggests that when women of childbearing age have abruptly decreased menstrual flow with increased testosterone, clinicians should pay attention to intra-ovarian occupying lesions and consider the possibility of LCT. In such cases, ultrasound examination can determine the presence, location, shape and size of occupying ovarian lesions and play an important role in the diagnosis of condition.

## 1. Introduction

Leydig cell tumor (LCT) is a rare type of sex cord-stromal tumors, accounting for no more than 0.1% of ovarian tumors.^[1,2]^ It is typically characterized by a tumor with endocrine function, and approximately 80% of patients with ovarian LCT have elevated serum testosterone and androstenedione levels.^[3]^

LCT is usually benign, and both clinical and laboratory markers of hyperandrogenemia can return to normal after surgical treatment, with an overall good prognosis. However, because of the very rare incidence of LCT and the few published case reports, gynecologists are usually unfamiliar with this type ovarian tumor. In addition, LCT may be asymptomatic and the ovarian masses in these patients are usually small and difficult to detect by gynecologic examination, which makes them difficult to diagnose. Ultrasound is a dynamic, real-time, noninvasive examination technique that is highly sensitive for the diagnosis of ovarian masses and is an effective tool for the evaluation of ovarian masses that can be widely used. We report a 38-year-old female with LCT whose gynecologic ultrasound suggested a persistent hypoechoic area in the right ovary with abundant blood flow around it. Our case is a useful addition to the diagnosis of LCT ultrasound in women of childbearing age.

## 2. Case report

A 38-year-old woman, G1P1, last menstrual period: 2022-05-05, used to have regular menstruation, 5 to 6/30 days, with moderate volume and no dysmenorrhea. Six months ago, there was a change in menstruation without any obvious trigger, the cycle became 10 to 45 days, the period was shortened to 0.5 to 1 day and the volume was very low. This study was approved by the First Hospital of Hebei Medical University, and informed written consent was obtained from the patient for publication of this case report.

**Figure 1.**
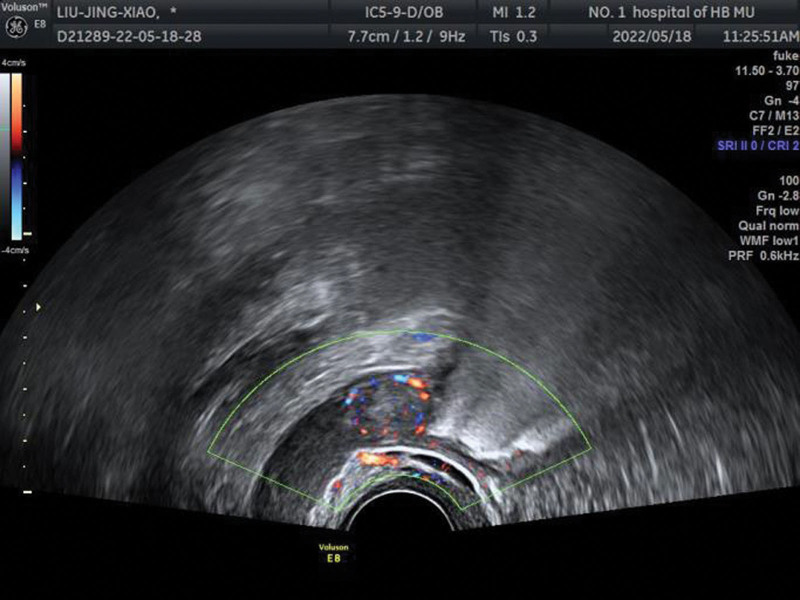
Transvaginal ultrasound: a hypoechoic area in the right ovary, with abundant blood flow around it.

Transvaginal ultrasound: the endometrial thickness was about 0.35 cm, and a hypoechoic area of approximately 1.49 × 1.20 cm in size was found in the right ovary, with abundant blood flow around it. On postmenstrual review: the endometrial thickness was about 0.37 cm and a hypoechoic area of about 2.03 × 1.52 cm in size with abundant blood flow around it were found in the right ovary (Fig. 1). No significant organic lesions were found on the pituitary MRI plain + enhancement scan. Abdominal CT plain + enhancement scan did not reveal any occupying adrenal lesion. CT of the chest showed no evidence of pleural effusion or pulmonary metastases.

Laboratory tests showed: testosterone: 7.17 ng/mL, estradiol: 151.55 pg/mL, progesterone: 0.18 ng/mL, follicle-stimulating hormone: 6.62 mIU/mL, luteinizing hormone: 1.46 mIU/mL. Cancer antigen 125, cancer antigen 199, alpha-fetoprotein and carcinoembryonic antigen were all within the normal range. Testosterone and dihydrotestosterone were elevated in the androgen metabolic pathway, and pregnenolone was decreased in the progesterone metabolic pathway, while other hormone concentrations were normal.

**Figure 2.**
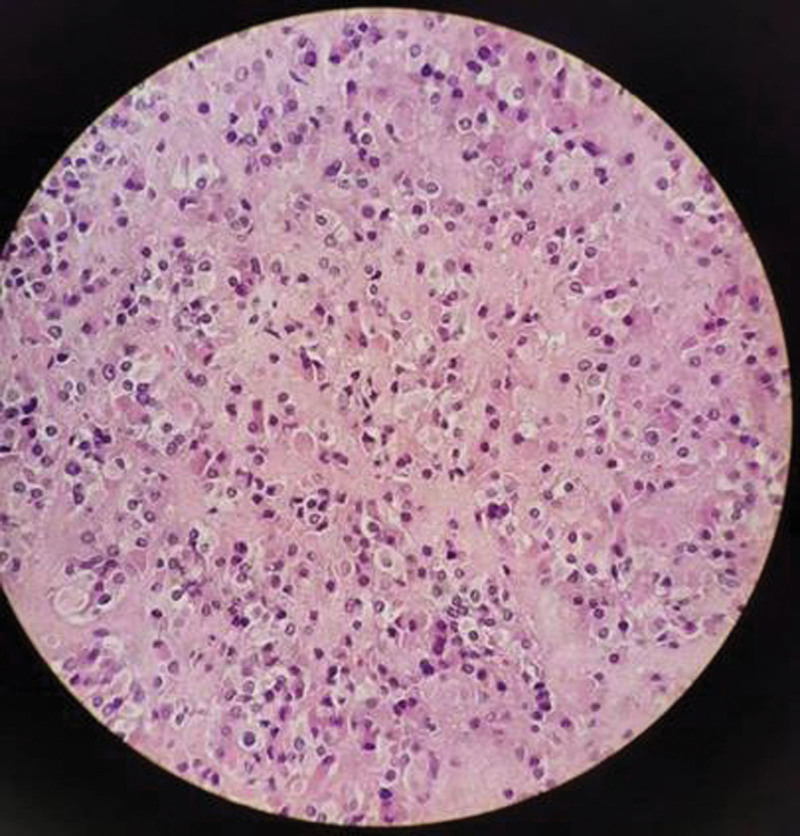
Pathological findings confirmed LCT in the 38-year-old woman’s right ovarian. LCT = Leydig cell tumor.

The patient’s abdominal CT plain + enhanced scan did not reveal an adrenal occupying lesion, and the transvaginal ultrasound suggested the presence of a hypoechoic area in the right ovary with elevated androgens, not excluding the possibility of interstitial tumor of the ovarian sex cord, so laparoscopic right adnexectomy was performed after obtaining the patient’s informed consent. Intraoperatively, a cyst of about 2.5 cm in diameter was seen in the right ovary, which was filled with dark yellow mucous material (Fig. 2). Postoperative pathology confirmed the morphology and immunohistochemistry of the right adnexa consistent with LCT, and no areas of malignant transformation were found on multiple sections of the surgical specimen. Immunohistochemical analysis showed positive for Inhibin-a, CR, Melan-A and CD56, weakly positive for Vimentin, Ki-67 proliferation index < 2%, negative for CK, WT1, SALL4(-) and CD10 (Fig. 2).

At the first follow-up visit 3 months after surgery, serum estradiol, luteinizing hormone and follicle-stimulating hormone levels returned to normal, with no signs of recurrence.

## 3. Discussion

LCT is a rare ovarian tumor belonging to a type of interstitial tumors of the ovarian sex cord,^[4]^ the etiology and pathogenesis of which are still unclear, the mass usually occurs unilaterally, is confined to the ovary and has a good prognosis.^[5,6]^ The pathology is classified as highly differentiated, moderately differentiated, hypofractionated, and containing heterologous components, and the degree of differentiation correlates with the patient’s prognosis.^[7]^ It provides a basis for early diagnosis because it is often associated with abnormal hormone levels. A review of the previous literature shows mostly case reports.

Since mesenchymal cell tumor is a unique functional ovarian tumor that produces testosterone, leading to hyperandrogenemia and masculinization in women, the salient feature of LCT is its endocrine function, with 75% of patients experiencing masculinization, including increased hair growth, seborrhea, acne, alopecia, hoarseness, decreased subcutaneous tissue deposits, breast atrophy, clitoral enlargement, menorrhagia and even amenorrhea.^[8,9]^ Very few patients have elevated estrogen levels and exhibit symptoms such as abnormal uterine bleeding, menstrual disorders, weight gain, endometrial hyperplasia, and endometrial polyps.^[10]^ The clinical signs and symptoms of patients are related to hormonal secretion or ovarian occupational lesions. Approximately 90% of patients can be detected in the early stages of the disease, with less than 3% presenting with extra-ovarian dissemination and a small number of patients with other combined ovarian lesions.^[11,12]^ Most patients have the opportunity to choose a surgical option that preserves reproductive function after early diagnosis, and testosterone levels generally decrease rapidly to normal after tumor removal and patients are able to regain their femininity quickly.^[5]^

Imaging ancillary examinations are very important in diagnosis, and ultrasonography is a noninvasive, convenient, efficient, and mature examination modality that has become the preferred method for adnexal tumor diagnosis because of its high sensitivity, good acceptability, and low cost.^[13]^ Ultrasonography is able to detect most ovarian masses and to monitor their changes dynamically, among which transvaginal ultrasound has a high sensitivity.^[7]^ Compared with abdominal ultrasound, the former can more clearly and accurately assess the size, location, and characteristics of ovarian masses because of the higher frequency of the probe and the closer proximity to the ovaries,^[13]^ making transvaginal ultrasound the best test for the initial evaluation of ovarian occupying lesions and to assist clinicians in better diagnosis of LCT. The typical ultrasound presentation of LCT is a solid, cystic or mixed mass, 60% of LCTs present as mixed masses on ultrasound and simple cysts are extremely rare. It is mostly a unilateral lesion, more rarely bilateral, with an intact envelope, well-defined from the periphery, small in size (<4 cm),^[2,8]^ and usually rich blood flow can be detected by ultrasound.^[9]^ The ultrasound presentation of this disease has been less reported in the previous literature. In this case, the ultrasound image suggested a persistent hypoechoic area in the right ovary with abundant blood flow around it, which was considered an ovarian lesion. This provided an important help for the clinician to determine the patient’s condition.

Of course, there are some limitations of ultrasonography which cannot identify lesions that are too small and has poor visualization of the subtle structures within the lesion, resulting in such masses can easily be missed or mistaken for a corpus luteum. Therefore, when the patient’s androgens are significantly higher than normal, even a normal ultrasound presentation cannot exclude the diagnosis of LCT. MRI has a high soft tissue resolution and is also meaningful for the detection of ovarian tumors when clinically indicated, but no abnormalities are seen on ultrasound.^[5]^ However, MRI is not preferred in clinical practice because of its high cost.

We reviewed previous case reports of LCT and found that LCT occurs mostly in postmenopausal women,^[2,14]^ with the appearance of masculine features as the main symptom. In this case, the patient was a female of childbearing age with the first symptom of significantly decreased menstrual flow. After excluding lesions of uterine, pituitary and hypothalamic origin, elevated testosterone and the persistence of the hypoechoic area in the right ovary suggested by ultrasound were important to clarify the patient’s diagnosis and treat the patient with early surgery, and postoperative pathology and immunohistochemistry confirmed that the case was consistent with LCT.

## 4. Conclusion

In conclusion, this case suggests that when women of childbearing age have abruptly decreased menstrual flow with increased testosterone, clinicians should pay attention to intra-ovarian occupying lesions and consider the possibility of LCT. Ultrasonography can help examine the presence, location, shape and size of occupying ovarian lesions, which can assist clinicians in diagnosing the condition and performing timely surgery to relieve the symptoms of hyperandrogenemia and help patients resume normal menstruation.

## Author contributions

CT contributed to the analysis and interpretation of data and revising of the manuscript for important intellectual content. XL was responsible for data curation. JL contributed to acquisition of additional clinicopathological data. YH was responsible for the acquisition of data, and drafting of the manuscript. ZF was responsible for final approval of the manuscript to be published. All authors contributed to and approved of the final version of the manuscript.

**Data curation:** Cai Tian, Xiaodong Li.

**Methodology:** Xiaodong Li.

**Project administration:** Jingqiao Liu.

**Writing – original draft:** Yiwei Han.

**Writing – review & editing:** Zijie Fu.

## References

[1] Higuchi A Tsuji S Amano T. Ovarian Leydig cell tumour diagnosis in a postmenopausal woman with uterine bleeding: a case report and literature review. J Obstet Gynaecol. 2022:1–3. 10.1080/01443615.2022.2027897 35164632

[2] Nardo LG Ray DW Laing I. Ovarian Leydig cell tumor in a peri-menopausal woman with severe hyperandrogenism and virilization. Gynecol Endocrinol. 2005;21:238–41. 16316848 10.1080/09513590500369005

[3] Prunty FT. Hirsutism, virilism and apparent virilism and their gonadal relationship. II. J Endocrinol. 1967;38:203–27. 5338183 10.1677/joe.0.0380203

[4] Lu Z Chen J. [Introduction of WHO classification of tumours of female reproductive organs, fourth edition]. Zhonghua bing li xue za zhi. 2014;43:649–50. 25567588

[5] Suturina LV Sharifulin EM Sharifulin MA. The Leydig Steroid Cell Tumor in a postmenopausal woman with clinical and biochemical hyperandrogenism: a case report. Metabolites. 2022;12:620. 35888744 10.3390/metabo12070620 PMC9320079

[6] Mourinho Bala N Aragüés JM Guerra S. Ovarian Leydig cell tumor: cause of virilization in a postmenopausal woman. Am J Case Rep. 2021;22:e933126. 34449760 10.12659/AJCR.933126 PMC8409458

[7] Abu-Zaid A Azzam A Alghuneim LA. Poorly differentiated ovarian sertoli-leydig cell tumor in a 16-year-old single woman: a case report and literature review. Case Rep Obstetr Gynecol. 2013;2013:858501. 10.1155/2013/858501 PMC3708428 23878752

[8] Chen M Zhou W Zhang Z. An ovarian Leydig cell tumor of ultrasound negative in a postmenopausal woman with hirsutism and hyperandrogenism: a case report. Medicine. 2018;97:e0093e0093. 29517680 10.1097/MD.0000000000010093 PMC5882447

[9] Fanta M Fischerová D Indrielle-Kelly T. Diagnostic pitfalls in ovarian androgen-secreting (Leydig cell) tumours: case series. J Obstet Gynaecol. 2019;39:359–64. 30428740 10.1080/01443615.2018.1517148

[10] Kozan P Chalasani S Handelsman DJ. A Leydig cell tumor of the ovary resulting in extreme hyperandrogenism, erythrocytosis, and recurrent pulmonary embolism. J Clin Endocrinol Metabol. 2014;99:12–7. 10.1210/jc.2013-3108 24152686

[11] Omo-Ogboi AC Deavers MT Schmeler KM. Collision tumor of the ovary involving sertoli-leydig cell tumor and high-grade serous carcinoma-report of the first case. Int J Gynecol Pathol. 2022. 10.1097/PGP.0000000000000896 35838626

[12] Ravichandaran A Lakshmanan A Kurian A. Moderately differentiated Sertoli-Leydig cell tumor of ovary with associated mucinous carcinoma and carcinoid—a case report and review of literature. Indian J Pathol Microbiol. 2021;64:528–31. 34341265 10.4103/IJPM.IJPM_672_20

[13] De Oliveira Franzin CM Kraft ML Faundes D. Detection of ovarian Sertoli-Leydig cell tumors exclusively by color Doppler sonography. J Ultrasound Med. 2006;25:1327–30. 16998106 10.7863/jum.2006.25.10.1327

[14] Klimek M Radosz P Lemm M. Leydig cell ovarian tumor-clinical case description and literature review. Prz Menopauzalny. 2020;19:140–3. 33100950 10.5114/pm.2020.99578 PMC7573336

